# Time Course of Cardiac Arrhythmia Following High‐Volume Exercise in Recreational Cyclists

**DOI:** 10.1161/JAHA.125.044378

**Published:** 2025-12-03

**Authors:** Daniel W. T. Wundersitz, Blake E. G. Collins, Brett A. Gordon, Carl J. Lavie, Jessica Orchard, Narelle Blair, Voltaire Nadurata, Michael I. C. Kingsley

**Affiliations:** ^1^ Holsworth Biomedical Research Centre, La Trobe Rural Health School La Trobe University Bendigo Victoria Australia; ^2^ John Ochsner Heart and Vascular Institute Ochsner Clinical School–The University of Queensland School of Medicine New Orleans LA USA; ^3^ Sydney School of Public Health The University of Sydney Sydney NSW Australia; ^4^ CardioScan Services Camberwell Victoria Australia; ^5^ Department of Cardiology Bendigo Health Bendigo Victoria Australia; ^6^ Department of Exercise Sciences, Faculty of Science University of Auckland Auckland New Zealand

**Keywords:** arrhythmias, cardiac, exercise, ions, physical endurance, Arrhythmias, Exercise, Electrophysiology

## Abstract

**Background:**

The aim of this study was to investigate the time course of cardiac arrhythmias following a bout of high‐volume exercise and to identify factors associated with abnormal cardiac arrhythmia.

**Methods:**

Thirty‐four recreational cyclists with 12 (interquartile range, 7–20) years of riding experience were recruited for this repeated‐measure cohort study. Participants cycled for up to 6 hours at ≈80% heart rate reserve. Cardiac arrhythmias were recorded using a 5‐lead Holter monitor for 4 days before exercise, the day of exercise, and 4 days after exercise. Arrhythmias were quantified as totals, abnormal arrhythmias (arrhythmias meeting international consensus criteria for cardiac arrhythmias requiring further investigation), and relative risk.

**Results:**

Sixty‐eight percent of participants displayed abnormal cardiac arrhythmias. No between‐day differences in the number of arrhythmias were found (*P*>0.10). However, the proportion of cyclists with abnormal ventricular arrhythmias was higher on the exercise day compared with preexercise days and the day after exercise (*P*<0.05). The relative risk of abnormal cardiac arrhythmia on the exercise day increased proportionally with the number of days that preexercise arrhythmias were recorded (peaking at a relative risk of 5.0 for those with arrhythmia on ≥3 preexercise days). Abnormal cardiac arrhythmia on the exercise day was associated with age, preexercise premature ventricular contractions, and preexercise abnormal cardiac arrhythmia.

**Conclusions:**

High‐volume cycling increased the likelihood of abnormal ventricular arrhythmia on the day of exercise. Extended monitoring revealed a high prevalence of abnormal cardiac arrhythmias among recreational cyclists. Current screening practices in many countries are typically limited to elite athletes, meaning recreationally active people are not routinely assessed for exercise‐induced arrhythmias. Our findings suggest that some individuals in this group may have clinically relevant arrhythmias that would otherwise go undetected without targeted evaluation.

**Nonstandard Abbreviations and Acronyms**
NSVT: nonsustained ventricular tachycardia

**Research Perspective**

**What Is New?**

- A bout of high‐volume cycling transiently increases the likelihood of abnormal ventricular arrhythmias in recreational cyclists, with elevated risk linked to preexisting ectopy and age.

**What Question Should Be Addressed Next?**

- Do repeated bouts of endurance exercise in recreational cyclists lead to long‐term cardiac remodeling or increased arrhythmia burden?

The benefits of regular exercise are well documented, with meaningful health gains achieved through modest engagement.^1^ Extensive research into the prognostic effect of exercise has led to the establishment of a minimal threshold, with higher amounts of exercise often inferred to provide additional health gains.^2^ Consequently, increasing numbers of people exceed exercise recommendations by 5‐ to 10‐fold^1^ and participate in high‐volume exercise events.^3^ High‐volume exercise increases cardiac output 5‐ to 7‐fold,^4^ eliciting potentially maladaptive structural changes to the heart, including increase of cardiac arrhythmias^5^, ^6^, ^7^ and, in rare cases, sudden cardiac death.^8^ In cases of sudden cardiac death, exercise is considered a potential trigger for underlying cardiovascular pathology, including malignant cardiac arrhythmias.^1^ While this research has been typically conducted in professional athletes, the increasing uptake of high‐volume exercise in the general population means that more recreationally active people with variable training backgrounds are being exposed to high‐volume exercise.

Cycling has increased in popularity among recreationally active people^9^ and is classified as a sport with the highest combination of static and dynamic components.^10^ Furthermore, among athletic populations, cyclists have the highest proportions of distinctly abnormal ECGs (35%; odds ratio [OR] 6.0 [1.2‐29.7])^11^ and risk for acute cardiac events, such as myocardial infarction (cyclists versus runners OR 3.1 [1.3‐5.0], p < 0.001).^12^ Although several studies have assessed high‐volume exercise and cardiac arrhythmia incidence,^13^, ^14^, ^15^, ^16^, ^17^, ^18^, ^19^, ^20^ only 1 investigated cyclists.^20^ Our earlier work^20^ showed that ventricular and atrial arrhythmias in adults increased after a multiday, high‐volume ride (odds ratio, [range 5.7–149.8]). However, this measurement, like all previous studies, was taken at a discrete and opportune time point (24‐hour monitoring ≈3 days after ride). Considering that many arrhythmias are evanescent,^21^ it can be argued that no previous research quantifies cardiac arrhythmias across an appropriate time period to identify transient changes before, during, and after high‐volume exercise.

To decrease the arrhythmia‐related risk of morbidity and sudden cardiac death during exercise, standards are needed to establish the importance of cardiac arrhythmias when they appear.^21^ International consensus criteria for ECG interpretation and evaluation classify numerous atrial (supraventricular tachycardia, atrial fibrillation/flutter) and ventricular (couplets, triplets, nonsustained ventricular tachycardia [NSVT]) arrhythmias as abnormal among athletes on a resting 12‐lead ECG.^22^ Former high‐volume athletes experience more ventricular (18%–23%) and atrial (62%–67%) arrhythmias than young athletes (≤4%) in elite populations.^23^ However, the rates of abnormal cardiac arrhythmias meeting these criteria before, during, and after exercise in people who are recreationally active remain unknown. Therefore, the aim of this study was to systematically investigate the time course of cardiac arrhythmia around a single bout of high‐volume cycling in recreationally active people. A secondary aim was to assess risk factors associated with abnormal arrhythmias that require further investigation according to international consensus criteria.

## Methods

### Data Availability Statement

Data underlying this article will be shared upon request to the corresponding author.

### Participants and Sample Size

Thirty‐four recreational cyclists (aged 52.0±12.7 years; height, 174±13 cm, weight, 78.7±12.6 kg; waist circumference, 88.8±9.2 cm; 70.6% men) provided written informed consent to participate in this study, which was approved by a Human Research Ethics Committee (AM/64246/BHCG‐2020‐245 005). Participants were eligible if they were aged ≥18 years, cycled ≥1 hour per week, and had no contraindications to exercise as determined by the Australian Adult Pre‐Exercise Screening System.^24^ All participants were medically cleared by their general practitioner to engage in high‐volume exercise, and no participant was excluded on the basis of health risk. Additional health and training history data were collected following the baseline visit. Participants had been cycling for a median of 12 (range, 1–50) years, covering 199 km (range, 23–630) per week, with a mean peak oxygen consumption of 42.9±9.7 mL/kg per min. As part of the Adult Pre‐Exercise Screening System screening, 5 participants reported any history of feeling faint, dizzy, or losing balance during exercise; 20 reported a family history of heart disease; 1 was on medication for high cholesterol and 2 on medication for high blood pressure; 1 reported smoking; all reported normal fasting blood glucose levels; and 7 were taking medications, none of which were associated with cardiac arrhythmias.

To identify a measurement day difference in the number of cardiac arrhythmias identified, a sample of 30 participants allowing for 20% dropout was required, with a correlation of 0.7 among repeated measures, an absolute effect size of 0.15 (small effect) when the α (*P*≤0.05) was corrected for repeated measurements and a power of 0.9 (GPower version 3.1.9.7; Kiel University, Germany). This study was conducted and reported in accordance with the Strengthening the Reporting of Observational Studies in Epidemiology statement.

### Procedures

**Figure 1:**
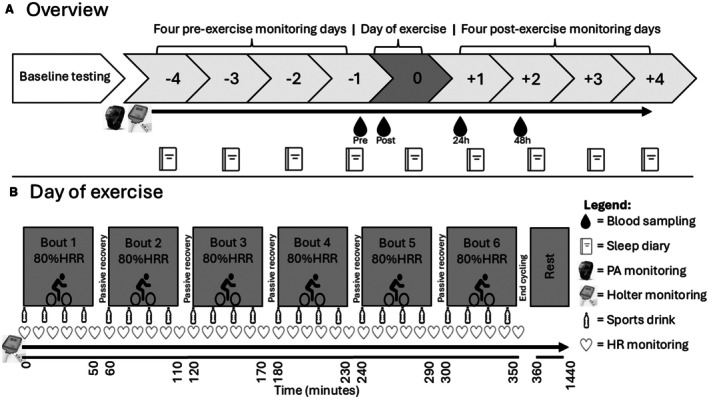
Study protocol timeline and detailed exercise day breakdown **A**, Overview of the 9‐day monitoring period, comprising 4 preexercise baseline days (−4 to −1), 1 exercise day (0), and 4 recovery days (+1 to +4). **B**, Detailed schedule of day 0, during which participants completed six 50‐minute cycling bouts, each separated by a 10‐minute passive recovery period. This was followed by ≈18 hours of continuous passive monitoring. HR indicates heart rate; HRR, heart rate reserve; and PA, physical activity.

We used a repeated‐measures cohort design with the same testing protocols and data collection across all visits to minimize bias and ensure consistency over time. Participants attended the laboratory on 6 occasions and completed 9 days of continuous ECG monitoring over the course of ≈2 weeks (Figure 1A). Baseline testing was completed during the initial laboratory visit, where participants completed paper‐based questionnaires (cycling history, 21‐item Depression Anxiety Stress Scale, Obstructive Sleep Apnea‐50) anthropometry, supine blood pressure, and arterial stiffness (Sphygmocor XCEL PWA/PWV; AtCor Medical Holdings Ltd, West Ryde, NSW, Australia), and a graded exercise test on an electromagnetic cycle ergometer (Excalibur Sport; Lode B.V., Groningen, Netherlands). During the graded exercise test, participants warmed up for 5 minutes at a self‐selected intensity then started cycling at a work rate of 100 W and increased in 25 W increments every minute until volitional exhaustion. A metabolic cart (TrueOne 2400; ParvoMedics, Salt Lake City, UT) was used to measure heart rate (Polar OY, Kempele, Finland) and oxygen uptake from expired gas, which were subsequently used to determine peak oxygen uptake and calculate the exercise intensity for the intervention. The first 18 participants then visited a local hospital cardiology department, where 12‐lead ECG (MAC 5500 HD, GE HealthCare, Chicago, IL) and transthoracic echocardiogram (General Electric Vivid E9; GE HealthCare) were performed. These investigations were not possible for all participants due to COVID‐19‐related restrictions that were periodically in place at the hospital.

Four days before the start of the exercise intervention (≥1 day after performing the graded exercise test) participants visited the laboratory to fit a 5‐lead Holter monitor (Medilog AR; Schiller, Miami, FL) sampling at 1000 Hz and an activity monitor (GT3X+; ActiGraph LLC, Pensacola, FL). ECG electrodes were placed on shaved, abraded, and cleaned skin at the fifth intercostal space in the midclavicular line, fifth intercostal space in the anterior axillary line (same level as V4), left arm over the deltoid fossa, and 2 placed on the central chest (tip of the manubrium and over the xiphoid process in line with sixth costal bone attachment) according to manufacturer guidelines. The Holter monitor was worn for the duration of the study and only temporarily removed on the exercise day to download control period data and when washing. Participants were instructed to refrain from undertaking additional exercise and structured physical activity and to wear the activity monitor on the nondominant wrist for the duration of the study. Each morning, they were also instructed to complete a customized sleep diary.

Participants returned to the laboratory (temperature controlled to 21 °C) on the day of exercise (Figure 1A) to cycle on the Excalibur Sport ergometer for up to 6 hours, completed in six 50‐minute exercise bouts interspersed with 10‐minute passive recovery (Figure 1B). The initial work rate was set at an intensity equivalent to ≈80% heart rate reserve^25^ derived from the graded exercise test and adjustments were made (as necessary) every 10 minutes according to the heart rate response (Polar OY). Exercise was stopped after 6 hours or if the participant reached volitional exhaustion. Rating of perceived exertion (20‐point Borg scale) was recorded during the initial and final minute of each 50‐minute exercise bout. A sports drink (Trace 72; Staminade, Villawood, NSW, Australia) was consumed at a rate of 0.7 g/kg per h of carbohydrate in an electrolyte solution^26^ every 15 minutes to favorably impact metabolic responses.

Venous blood samples were taken before exercise, immediately (<10 minutes) after exercise, and at 24 and 48 hours after exercise according to standard phlebotomy procedures using a 19G needle (BD Medical, North Ryde, NSW, Sydney) inserted into the medial antecubital vein. Blood samples were collected into a 10‐mL serum separating tube, a 10‐mL fluoride oxalate tube, a 5‐mL EDTA‐treated tube, and two 5‐mL sodium citrate tubes. The serum separating and fluoride oxalate tubes were stored on ice and taken to a commercial pathology laboratory (Australian Clinical Laboratories, Bendigo, Victoria, Australia) for clinical analyses. Analyses included high‐sensitivity cardiac troponin, high‐sensitivity C‐reactive protein, BNP (B‐type natriuretic peptide), calcium, chloride, magnesium, potassium, and sodium. After hematocrit and hemoglobin concentrations were analyzed as described previously^27^ and used to correct postexercise blood plasma volumes,^28^ an ELISA kit was used to determine BNP concentration (Human BNP, ab193694; Abcam, Melbourne, Australia) from sodium citrate plasma samples.

### Holter and Activity Data Analyses

Holter data were analyzed by a cardiac technician using commercial software (Darwin V2; Medilog, Schiller AG, Baar, Switzerland) and visually verified by cardiologist blinded to timing of the intervention. Cardiac arrhythmias included premature ventricular contraction (PVC), ventricular/atrial couplet, ventricular/atrial triplet, NSVT, idioventricular rhythm, premature atrial contraction, supraventricular tachycardia, and atrial fibrillation/flutter. International consensus criteria for interpreting ECGs in athletes suggestive of underlying pathology were used to define abnormal cardiac arrhythmias.^22^ Specifically, the following occurrences were classified as abnormal cardiac arrhythmia: (1) atrial arrhythmias (supraventricular tachycardia, atrial fibrillation/flutter) and (2) ventricular arrhythmias (ventricular couplets, ventricular triplets, NSVT). Premature atrial contraction and PVC burden was considered high if it exceeded 10% of all cardiac cycles.^29^ Participants who experienced an abnormal arrhythmia were notified of their results, and it was suggested that they visit their general practitioner for follow‐up (with data made available upon request).

Activity data were analyzed using manufacturer software (Actilife version 6.13.6; ActiGraph LLC, Pensacola, FL). Vector magnitude data were calculated in 60‐second epochs and expressed as mean counts per minute. The Choi algorithm was used to determine nonwear time. Cut points of <2860 counts per minute (sedentary), 2860 to 3940 counts per minute (light physical activity) and >3941 counts per minute (moderate to vigorous physical activity) were used on the basis of wrist‐worn monitoring in adults.^30^

### Statistical Analysis

All statistical analyses were performed using SPSS statistics version 29 IBM Corporation, Armonk, NY). Statistical significance was set at *P*≤0.05. Normality was assessed using Shapiro‐Wilk tests (*P*>0.05) and visual inspection of histograms. Data were expressed as mean and 95% CI or median (interquartile range) if the distribution breached the assumption of normality.

Cochran’s Q test was performed to determine whether the proportion of participants who displayed abnormal cardiac arrhythmias (dichotomous outcome) changed with high‐volume exercise by day (within factor: measurement time=9 days; control [n=4], exercise [n=1], and after exercise [n=4]). Post hoc testing was performed using multiple McNemar’s tests. Independent sample *t* tests (or nonparametric Mann–Whitney *U* test, where appropriate) were used to compare the risk profiles of participants with or without abnormal cardiac arrhythmias, as defined by international consensus criteria.^22^ Variables assessed included age; number of PVC’s; training history; ionic blood markers; Depression, Anxiety and Stress Scale scores; fitness; family history of heart disease; and Obstructive Sleep Apnea‐50 score.

To assess the potential influence of undiagnosed coronary artery disease on study outcomes, sensitivity analyses were conducted by comparing participants who underwent 12‐lead ECG testing (n=11) with those who did not (n=23). Two independent sample *t* tests were performed to evaluate differences in the proportion of participants exhibiting abnormal cardiac arrhythmias, 1 focused on the exercise day alone and the other across the full monitoring period.

To explore whether preexisting arrhythmia patterns influenced the likelihood of abnormal arrhythmias during exercise, relative risk was calculated. The relative risk of abnormal cardiac arrhythmia on the exercise day was computed for the people who had abnormal cardiac arrhythmia on (1) ≥1, (2) ≥2, and (3) ≥3 preexercise days with reference to people who had no arrhythmia on any of the preexercise days (where the relative risk=1.0).

To evaluate the effects of high‐volume exercise on blood biomarkers and physical activity levels, 1‐way repeated‐measures ANOVAs (or nonparametric Wilcoxon signed‐rank tests for variables with 2 time points and Friedman’s test for variables with ≥3, where appropriate) were conducted for variables measured at multiple time points. Post hoc pairwise analyses were conducted with Bonferroni corrections where necessary.

## Results

Twenty‐four participants completed all six 50‐minute exercise bouts, and 33 participants completed at least 5 bouts (the last reached volitional exhaustion 10 minutes into the fourth bout). Participants cycled for 288±26 minutes at an average power output of 137±34 W (starting intensity, 142±32 W; final intensity, 128±33 W). Average heart rates were 68±9 beats per minute before exercise and 138±14 beats per minute during exercise. The exercise heart rate corresponded to 93% (95% CI, 91%–96%) of the target 80% heart rate reserve threshold.

Perceived exertion (rate of perceived exertion) after the first hour was 11±2, increasing by ≈1 unit each hour, reaching 15±2 during bouts 5 and 6. Participants consumed 0.80±0.17 L of sports drink per hour, totaling 4.82±1.01 L over the session.

### Cardiac Arrhythmias

**Figure 2:**
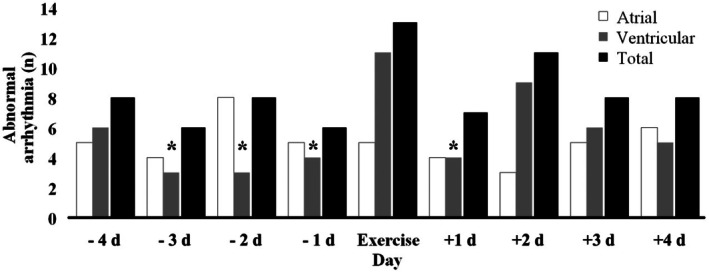
Daily frequency and proportion of abnormal cardiac arrhythmias meeting international consensus criteria^22^ Data are presented as the binary count of participants experiencing arrhythmias (yes=1, no=0). Ventricular arrhythmias included couplets, triplets, and nonsustained ventricular tachycardia; atrial arrhythmias included paroxysmal supraventricular tachycardia and atrial fibrillation/flutter. Cochran’s *Q* test was used to assess changes in arrhythmia occurrence across the study timeline. A significant variation was observed for ventricular arrhythmias (*P*=0.036), but not for atrial (*P*=0.561) or total arrhythmias (*P*=0.232). *Ventricular arrhythmia proportion was significantly lower compared with exercise day (*P*<0.05).

A total of 28 196 142 normal cardiac cycles and 278 632 (0.99%) cardiac arrhythmias were captured over 9 days of monitoring (Table 1). Sixty‐eight percent of participants displayed abnormal cardiac arrhythmias on at least 1 day (Table 2).^22^ The proportion of participants displaying abnormal cardiac arrhythmias did not differ by day (χ^2^_8_=10.50, *P*=0.232; Figure 2).^22^ However, the day‐to‐day proportion of participants with abnormal ventricular arrhythmias changed over time (χ^2^_8_=16.49, *P*=0.036), being higher on the exercise day compared with most preexercise days (−3 to −1) and the first day after exercise (*P*<0.05). The proportion of participants with abnormal atrial arrhythmias were similar on all days (χ^2^_8_=6.78, *P*=0.561). Of the 13 participants who displayed abnormal rhythms on the exercise day, 4 experienced these before exercise, 6 during exercise, and 9 following exercise.

**Table 1:** Cardiac Arrhythmia Events and Proportions by Monitoring Period and Cumulative Total Over 9 Days

	Preexercise daily average*		Exercise day		Postexercise daily average*		Total (%)	
	Number	Percentage	Number	Percentage	Number	Percentage	Number	Percentage
Ventricular								
Premature ventricular contraction	18 053	97	13 810	91	14 153	97	149 688	100
Low burden (≤10%)	2670	97	1966	97	1314	97	17 909	97
High burden (>10%)	15 383	3	11 844	3	12 837	3	131 779	3
Ventricular couplet	3.3	18	33	26	16.8	35	112	50
Ventricular triplet	1	12	1	3	0.5	3	7	15
NSVT, beats	1	9	3	6	1.8	15	14	24
4–5	0.5	6	2	6	1.2	12	10	15
6–10	0.3	3	0	3	0.25	3	2	6
>10	0	0	1	3	0.25	3	2	6
Idioventricular rhythm	0.3	3	0	0	1.5	12	7	15
Atrial								
Premature atrial contraction	13 816	100	11 433	91	13 448	97	120 490	100
Low burden (≤10%)	2878	97	11 433	100	2371	97	23 462	97
High burden (>10%)	10 938	3	0	0	11 077	3	97 028	3
Atrial couplet	50.3	47	72	24	58.3	47	506	62
Atrial triplet	10.8	29	156	26	9	29	235	50
Atrial tachycardia/SVT	11.5	29	18	15	38.3	32	214	41
Paroxysmal atrial fibrillation/flutter (≥30 s)	8	6	1	3	0	0	31	6
Pauses								
Pause ≥2 s	1142	32	567	15	335	24	6475	35
Pause ≥3 s	0	0	0	0	0.5	6	2	6

Data are presented as the total number of arrhythmias per day and the percentage of participants affected. No significant differences were observed in the number or type of cardiac arrhythmias by day (*P*≥0.107); therefore, all pre‐ and postexercise arrhythmias were pooled and averaged per day for analysis. Day‐to‐day differences did not reach statistical significance (*P*≥0.057). NSVT indicates nonsustained ventricular tachycardia; and SVT, supraventricular tachycardia.

* For comparison with the exercise day, pre‐ and postexercise values represent the total number of arrhythmias divided by 4, reflecting the 4 days of data in each period vs 1 on the exercise day.

**Table 2:** Risk Profile of Participants With or Without Abnormal Cardiac Arrhythmia Meeting International Consensus Criteria^22^

	Exercise day		Overall	
	Normal	Cardiac arrhythmia	Normal	Cardiac arrhythmia
	n=21	n=13	n=11	n=23
Sex, male, n (%)	15 (71)	9 (69)	8 (72)	16 (70)
Age, y (95% CI)	48.5 (42.3 to 54.7)	57.7 (52.5 to 63.0)*	45.9 (37.1 to 54.8)	55.0 (50.0 to 60.0)*
Weight, kg (95% CI)	80.0 (74.4 to 85.6)	77.3 (69.7 to 85.0)	78.9 (68.8 to 89.0)	79.1 (74.2 to 83.9)
Height, m (IQR)	1.80 (1.72 to 1.82)	1.75 (1.62 to 1.81)	1.76 (1.70 to 1.82)	1.74 (1.68 to 1.80)
Peak oxygen consumption, mL/kg per min (95% CI)	44.8 (40.3 to 49.3)	39.7 (34.4 to 45.1)	43.8 (37.5 to 50.2)	42.4 (38.1 to 46.7)
Current training, km/week (95% CI)	188.8 (127.2 to 250.4)	218.7 (136.3 to 301.2)	168.0 (92.3 to 243.6)	215.7 (153.6 to 277.8)
Training history, y (IQR)	18.0 (7.0 to 40.0)	10.0 (4.0 to 19.2)	18.0 (10.0 to 50.0)	15.8 (5.0 to 20.0)
Family history of heart disease, n (%)	9 (43)	5 (39)	7 (64)	13 (57)
OSA 50 Score ≥5, n (%)	11 (52)	8 (62)	5 (46)	14 (61)
DASS ≥mild depression	1 (5)	0 (0)	1 (9)	0 (0)
DASS ≥mild anxiety	3 (14)	1 (8)	1 (9)	3 (13)
DASS ≥mild stress	3 (14)	1 (8)	1 (9)	3 (13)
Preexercise arrhythmia, 1+ d, n (%)	5 (24)	9 (69)*	NA	14 (61)*
Preexercise PVC, n (IQR)	1 (1 to 2)	11 (4 to 83)*	2 (1 to 2)	11 (4 to 83)*
	Pre‐ to postexercise change in biochemical markers			
High‐sensitivity cardiac troponin, ng/L (95% CI)	12.6 (5.3 to 19.8)	10.4 (2.1 to 18.6)	9.4 (1.5 to 17.2)	12.9 (5.8 to 20.0)
B‐type natriuretic peptide, pg/mL (IQR)	2.42 (−17.2 to 26.8)	0.53 (−37.0 to 17.3)	6.36 (−49.0 to 30.4)	1.16 (−19.4 to 14.9)
Sodium, mmol/L (95% CI)	1.76 (−4.47 to 7.98)	3.24 (−3.41 to 9.89)	0.98 (−7.17 to 9.12)	2.91 (−2.80 to 8.62)
Magnesium, nmol/L (95% CI)	−0.01 (−0.06 to 0.04)	−0.002 (−0.07 to 0.07)	−0.00 (−0.08 to 0.08)	−0.01 (−0.06 to 0.04)
Calcium, mmol/L (95% CI)	0.02 (−0.09 to 0.14)	0.06 (−0.04 to 0.17)	0.01 (−0.15 to 0.18)	0.05 (−0.04 to 0.14)
Potassium, mmol/L (95% CI)	−0.04 (−0.42 to 0.34)	−0.07 (−0.59 to 0.45)	0.23 (−0.22 to 0.68)	−0.19 (−0.56 to 0.19)
Hematocrit, % (95% CI)	−0.11 (−1.57 to 1.36)	−0.61 (−1.85 to 0.63)	−0.34 (−2.27 to 1.59)	−0.26 (−1.54 to 1.01)
High‐sensitivity C‐reactive protein, 24 h change, mmol/L (95% CI)	8.91 (6.57 to 11.25)	9.29 (4.81 to 13.77)	8.51 (6.09 to 10.92)	9.30 (6.43 to 12.18)

	Echocardiographic characteristics			
	n≤10	n≤8	n≤6	n≤12
Ejection fraction, %	55.9 (52.5 to 59.3)	60.0 (55.2 to 64.9)	56.5 (50.2 to 62.8)	58.3 (54.9 to 61.8)
Interventricular septal diameter at end‐diastole, mm/m^2^	4.98 (4.57 to 5.39)	4.48 (3.78 to 5.17)	4.71 (4.18 to 5.24)	4.78 (4.25 to 5.31)
Left ventricular internal diameter at end‐diastole, mm/m^2^	25.3 (23.8 to 26.9)	25.0 (21.9 to 28.0)	24.9 (22.4 to 27.4)	25.3 (23.3 to 27.3)
Left ventricular internal diameter at end‐systole, mm/m^2^	16.9 (16.0 to 17.9)	16.3 (13.9 to 18.8)	17.0 (15.8 to 18.1)	16.5 (14.9 to 18.1)
Left ventricular mass, g/m^2^	124.1 (101.8 to 146.5)	109.1 (91.3 to 127.0)	106.7 (80.2 to 133.2)	121.4 (104.0 to 138.9)
Posterior wall thickness at end‐diastole, mm/m^2^	4.74 (4.26 to 5.23)	4.68 (4.05 to 5.30)	4.42 (3.75 to 5.09)	4.83 (4.39 to 5.28)
Right ventricle basal diameter in diastole, mm/m^2^	20.4 (18.4 to 22.3)	21.4 (19.1 to 23.6)	19.9 (16.8 to 22.9)	21.3 (19.6 to 22.9)
Right atrial volume, mL/m^2^	27.4 (22.8 to 32.0)	31.1 (21.9 to 40.2)	27.6 (21.1 to 24.0)	29.3 (23.8 to 34.9)

DASS indicates Depression, Anxiety and Stress Scale; IQR, interquartile range; OSA, Obstructive Sleep Apnea; and PVC, premature ventricular contraction.

* Cardiac arrhythmia value was significant compared with normal value (*P*<0.05).

**Figure 3:**
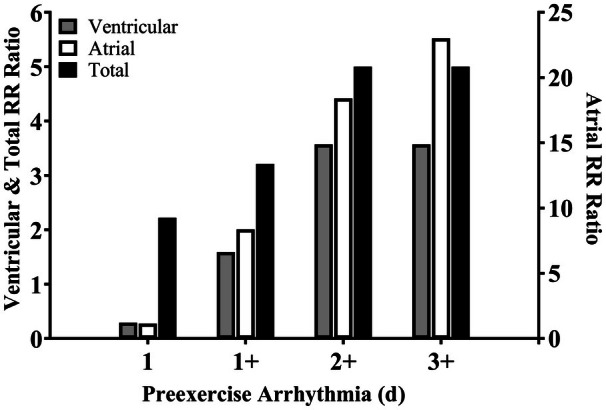
Relative risk of abnormal cardiac arrhythmia on the exercise day in relation to pre‐exercise arrhythmia occurrence The figure examines whether preexisting arrhythmia patterns affect the likelihood of experiencing abnormal arrhythmias during exercise, using RR ratios. The RR was calculated for individuals who had abnormal cardiac arrhythmia (as defined by international consensus criteria^22^) on (1) ≥1, (2) ≥2, and (3) ≥3 preexercise days, compared with those with no arrhythmias on any preexercise day (where the RR=1.0). RR indicates relative risk.

Relative risk of abnormal cardiac arrhythmia on the exercise day was 2.2 (95% CI, 0.7–7.0; *P*=0.170) for participants who displayed abnormal arrhythmia on ≥1 day before exercise, 3.2 (95% CI, 1.2–8.4; *P*=0.020) for ≥2 days, and 5.0 (95% CI, 2.1–12.0; *P*<0.001) for ≥3 days (Figure 3).^22^ A consistent trend was observed for ventricular and atrial arrhythmias. Age and the number of preexercise PVCs and abnormal cardiac arrhythmias displayed on ≥1 day before exercise were higher in participants who displayed abnormal cardiac arrhythmias (*P*≤0.05; Table 2). No echocardiographic measure differed between participants who exhibited abnormal cardiac arrhythmia and those who did not (*P*>0.157).

### Blood Biomarkers and Physical Activity Levels

High‐sensitivity cardiac troponin concentration increased from before exercise to after exercise (χ^2^_1_=27.0, *P*<0.001; Table 3). High‐sensitivity C‐reactive protein displayed a main effect for time (χ^2^_3_=87.27, *P*<0.001), with 24 and 48 hours after exercise elevated (*Z*>5.09, *P*<0.001). Glucose displayed a main effect for time (F_3,34_=18.52, *P*<0.001), with after exercise elevated (*P*<0.01). Calcium displayed a main effect for time (F_3,34_=3.97, *P*<0.043); however, no pairwise differences were found (*P*>0.144). All other analyses were not different from before exercise (*P*≥0.066).

**Table 3:** Physical Demand and Ionic Blood Marker Responses Before, After, and 24 to 48 Hours After Exercise

	Before exercise	After exercise	24 h after exercise	48 h after exercise
B‐type natriuretic peptide, pg/mL (IQR)†	138 (123–308)	141 (112–327)	…	…
High‐sensitivity cardiac troponin, ng/L (IQR)‡	4.0 (3.0–9.0)	15.0 (8.4–29.9)*	…	…
High‐sensitivity C‐reactive protein, mg/L (IQR)	0.35 (0.10–0.80)	0.45 (0.20–0.90)	8.65 (6.10–11.93)*	5.50 (3.45–7.43)*
Calcium, mmol/L (95% CI)	2.38 (2.36–2.41)	2.44 (2.37–2.51)	2.38 (2.35–2.41)	2.36 (2.34–2.38)
Chloride, mmol/L (95% CI)	105 (104–106)	105 (102–108)	105 (104–106)	106 (105–107)
Magnesium, mmol/L (95% CI)	0.82 (0.80–0.84)	0.81 (0.78–0.84)	0.84 (0.82–0.86)	0.82 (0.80–0.84)
Potassium, mmol/L (95% CI)	4.68 (4.54–4.83)	4.80 (4.58–5.02)	4.63 (4.52–4.74)	4.65 (4.49–4.81)
Sodium, mmol/L (IQR)	141 (140–143)	147 (136–150)	141 (140–142)	141 (140–143)
Glucose, mmol/L (95% CI)	4.71 (4.35–5.08)	6.36 (5.93–6.80)*	4.95 (4.55–5.35)	4.89 (4.51–5.27)

Values are either mean (95% CI) or median and lower to upper IQR depending on the distribution of the data. IQR indicates interquartile range.

* Value is significantly different from preexercise values (*P*<0.01).

^†^ B‐type natriuretic peptide values are based on 30 participants’ data due to pairwise comparisons being below detectable results.

^‡^ High‐sensitivity cardiac troponin values are based on 27 participants’ data due to commercial laboratory error.

Participants wore activity monitors for 21.4±0.4 hours per day with no difference in sedentary time by day (F_8,256_=1.389, *P*=0.242; Table S1). Although the main effect for light‐intensity physical activity revealed day‐to‐day differences (F_8,256_=2.64, *P*=0.027), the pairwise comparisons revealed no differences between days (*P*≥0.055). As anticipated, ambulatory moderate to vigorous physical activity was lower during the exercise day, which included up to 6 hours of exercise performed on a stationary cycle ergometer, in comparison with most days before and after exercise (χ^2^_8_=20.39, *P*=0.009).

### Hospital‐Based Cardiovascular Testing

Twelve‐lead ECG and 2‐dimensional echocardiogram data were available for 18 participants (see Tables S2 and S3). No evidence to suggest coronary artery disease was evident in any of the 12‐lead ECGs recorded (n=11; Table S4). Furthermore, when coronary artery disease was included as a covariate in the analysis, there were no significant differences in the proportion of participants exhibiting abnormal cardiac arrhythmias either on the exercise day (*t* [32]=−1.15, *P*=0.881) or across the full monitoring period (*t* [32]=−1.12, *P*=0.272).

## Discussion

The primary aim of this study was to assess the time course of cardiac arrhythmia surrounding a bout of high‐volume exercise in recreationally active cyclists. The main findings were (1) high‐volume exercise increased the proportion of participants experiencing abnormal ventricular arrhythmias compared with nonexercise days; (2) a substantial proportion of participants (68%) met international consensus criteria^22^ for arrhythmias warranting follow‐up; and (3) the likelihood of experiencing abnormal arrhythmias on the exercise day was associated with participant age, the number of preexercise PVCs, and the presence of abnormal arrhythmias on ≥1 preexercise day.

The proportion of participants experiencing ventricular arrhythmias was elevated on the exercise day when compared with most preexercise days, before returning to preexercise rates the day after exercise (Figure 2). Comparison with previous work investigating the time course of exercise‐induced ventricular arrhythmias is difficult, given that the findings are mixed across studies with variable collection periods and exercise modes.^13^, ^14^, ^15^, ^16^, ^17^, ^18^, ^19^, ^20^ Further, assessment protocols and outcomes have lacked standardization, where PVCs, ventricular flutter/fibrillation,^13^ NSVTs,^17^ and couplets^20^ have been identified using different monitoring methods. For example, D’Ascenzi et al^19^ reported no pre‐ to postmarathon difference in PVCs using short‐term 12‐lead ECG recordings, while Herm et al^17^ completed Holter monitoring from ≈72 hours before to ≈58 hours after a marathon and reported NSVT in 9.4% of participants during the marathon (data not presented for other time points). Nevertheless, physiological^31^ and muscular^10^ demands of high‐volume cycling facilitate elevated ventricular pressures,^32^ which, when combined with right ventricle dysfunction,^33^ has the potential to cause clinically significant ventricular arrhythmias. While this mechanism is supported by existing literature, our study did not include measures of cardiac function during exercise and therefore does not evaluate these mechanisms.

Given the potential association between risk of arrhythmia and ventricular dysfunction, an important finding from this study was that 68% of participants experienced self‐resolving but abnormal cardiac arrhythmias during the 9‐day monitoring period. This proportion is higher than previously reported in the research relating to high‐volume running (0%–16.8%),^13^, ^14^, ^15^, ^17^, ^18^, ^19^ skiing (22%),^16^ and cycling (27%).^20^ It is possible that the higher prevalence of abnormal arrhythmias in this study might be explained by the extended monitoring period in comparison with previous research. However, the rates identified on any single day (ie, 18%–38%) are broadly comparable (eg,^16^, ^17^, ^20^). In addition, 50% of participants in the current study displayed couplets, whereas Herm et al^17^ did not report couplets or triplets as being abnormal.^22^ Thus, for comparability, it is important that future work reporting cardiac arrhythmias in response to exercise adheres to current reporting standards and monitors participants over an appropriate temporal canvas. It is likely that longer‐term monitoring of athletes (recreational to professional) will identify a higher prevalence of abnormal cardiac arrhythmias than currently reported in the literature. Regardless, rates of abnormal cardiac arrhythmia are high in older recreational cyclists, suggesting that further screening of recreational active cyclists performing high‐volume exercise might be appropriate. As current international screening standards are designed for 12‐lead ECG data, Holter‐specific screening standards for athletic populations are warranted that account for the extended time of monitoring.

In line with previous research, the risk of abnormal cardiac arrhythmia on the exercise day was associated with age^17^, ^23^ and number of PVCs before exercise.^17^ Abnormal arrhythmias were further associated with presence of cardiac arrhythmias on ≥1 day before exercise (Table 3). Therefore, multiday preparticipation screening of older athletes undertaking, or planning to undertake, high‐volume exercise might be necessary to ensure that those at risk of exercise‐induced abnormal cardiac arrhythmia are identified. This finding reflects the transient nature of these arrhythmias and consequently the variability in preexercise arrhythmia, which occurred while participants completed consistent amounts of background ambulatory physical activity (see Table S1). Although the addition of longer‐term Holter monitoring would increase the burden to participants and clinicians at the current time, advances in wearable sensors and artificial intelligence (eg, Ding et al^34^) might make multiday screening viable for a large proportion of the population within a short time.

Several plausible and perhaps complementary mechanisms have been suggested to lead to a proarrhythmic state with high‐volume exercise performance including ionic fluctuations,^35^ right ventricle dysfunction,^36^ increased reactive oxygen species production,^37^ changes in pH, increased secretion of catecholamines,^38^ and vagal to sympathetic predominance.^13^ For example, ionic currents (from subcellular to organ) initiate various complex excitation and contraction dynamics that when disrupted in the ventricles may potentiate the risk of arrhythmia.^35^ Intracellular calcium cycling,^39^ dysregulation of intracellular sodium,^40^ and altered extracellular potassium^35^ in particular all play critical roles in cardiac arrhythmias. Given that alterations in these ions have been associated with arrhythmogenesis, participants consumed a sports drink containing electrolytes with the aim to maintain hydration and electrolyte balance. Plasma concentrations of calcium, chloride, magnesium, potassium, and sodium remained consistent throughout the study, demonstrating that change in abnormal ventricular arrhythmia on the exercise day was not mediated by change in these electrolytes at a systemic level. The right ventricle has also been implicated as a potential contributor to exercise‐induced arrhythmogenesis. The right ventricle is uniquely susceptible to hemodynamic stress during endurance activities such as cycling, due to its thinner wall and limited compliance.^41^ While our resting 2‐dimensional echocardiographic data did not show structural differences between participants with and without arrhythmias, previous data suggest that right ventricle dysfunction might become apparent only under exercise conditions.^36^ Dynamic assessments, such as exercise echocardiography or strain imaging, might be required to detect subtle impairments in right ventricle compliance that contribute to arrhythmia risk.^42^ Nevertheless, the acute mechanisms responsible for high‐volume exercise‐induced arrhythmia remain unclear.

The increase in abnormal ventricular arrhythmias across the exercise day is of some concern, especially as recreationally active people from the general population have a higher prevalence of cardiovascular disease risk factors than professional athletes.^43^ Prior researchers have shown that PVCs, even in asymptomatic individuals without structural heart disease, might carry prognostic significance.^44^ Notably, meta‐analyses have demonstrated that exercise‐induced ventricular arrhythmias, especially those occurring during the postexercise recovery phase, as observed in the participants of this study, have been associated with increased cardiovascular death.^45^, ^46^ The 2024 Heart Rhythm Society Expert Consensus Statement on Arrhythmias in the Athlete reinforces this concern, highlighting that certain ventricular arrhythmias, including high PVC burden and complex ectopy during or after exercise, may indicate elevated risk and warrant further evaluation, even in athletic populations.^29^ Further, ventricular arrhythmias remain an important cause of morbidity and account for nearly half of unexpected sudden cardiac death.^47^ These previous findings underscore the potential value of further evaluating such arrhythmias in recreationally active populations, where routine cardiac screening is uncommon, and symptoms, when present, may be misattributed to normal exertion. However, the clinical relevance of short, self‐limiting episodes of complex ventricular ectopy remains uncertain. In highly trained athletes, such findings might reflect benign physiological adaptations rather than pathology.^48^ While increased access to continuous monitoring may facilitate early detection of malignant arrhythmias, it also raises the risk of false positives, which can lead to medical, psychological, and financial consequences for individuals and health care systems. Future longitudinal studies are essential to clarify the prognostic value of these arrhythmias and to guide evidence‐based screening strategies that balance early detection with the risk of overtreatment. To support this, future studies should incorporate long‐term follow‐up of participants with identified arrhythmias to better understand their clinical relevance and inform appropriate screening and management strategies in recreationally active populations.

The relationship between exercise and arrhythmia incidence is complex, and the underlying mechanisms remain unclear. This complexity is compounded by the interrelated nature of exercise prescription variables, such as intensity, volume, duration, and type, which may contribute independently or synergistically to arrhythmogenic risk. This distinction is further complicated because high‐volume exercise at an absolute intensity that can be classified as being moderate might functionally resemble high intensity after being performed for a prolonged duration. In addition, these effects likely vary depending on individual susceptibility and cardiac phenotype. For example, previous research has linked acute high‐volume performance,^20^ chronic high‐volume endurance training,^49^, ^50^ higher race performance,^7^ lifetime accumulation of ≥2000 hours,^51^ and both short‐ and long‐duration high‐intensity training (long being the strongest marker)^52^ as being arrhythmogenic, whereas low‐,^52^ moderate‐, and high‐intensity interval training may reduce arrhythmia risk,^50^, ^53^ particularly when compared with sedentary individuals or those engaging in chronic high‐volume training. These findings highlight the challenge of disentangling volume from intensity in endurance exercise and suggest that arrhythmogenic risk might arise from their combined effects rather than either factor alone. A more nuanced understanding of these interactions is needed to inform safe and individualized exercise prescription.

There were several limitations in the current study, including the small sample size and the potential for recruitment bias, which may have existed due to the significant time commitment for participants. Most participants were men (71%) and could be classified within the masters athlete age group (91% aged >35 years), which limits the generalizability of results to broader recreationally active populations. Additionally, the lack of objective measures, such as baseline ECGs, for all participants means that underlying cardiovascular disease cannot be ruled out for some participants.

In this small cohort of predominantly male recreational cyclists, 9 days of monitoring around a single bout of high‐volume cycling identified a high rate of abnormal cardiac arrhythmias in a population that is rarely assessed. The proportion of participants who experienced abnormal ventricular arrhythmias was elevated on the day of exercise, suggesting a potential link between high‐volume cycling and acute arrhythmic risk. Given that current screening practices are typically limited to elite athletes, these findings highlight a gap in the routine evaluation of recreationally active individuals, who may also be at elevated risk of abnormal arrhythmias. Larger‐scale studies are warranted to determine the true prevalence and clinical significance of exercise‐induced arrhythmias in this population.

## Sources of Funding

This work was supported by the Bendigo Tertiary Education Anniversary Foundation through the Holsworth Biomedical Research Centre, and La Trobe University’s Sport, Exercise and Rehabilitation Focus Area–Grant Ready Scheme. Dr Wundersitz and Dr Collins are funded via the Bendigo Tertiary Education Anniversary Foundation, which is administered through the Holsworth Biomedical Research Centre. Professor Kingsley also receives salary support from the Bendigo Tertiary Education Anniversary Foundation. No direct external funding was provided for the conduct of this specific research project.

## Disclosures

None.

## Supporting information

Tables S1–S4
