# Supplementary material for: Time Course of Cardiac Arrhythmia Following High‐Volume Exercise in Recreational Cyclists
Source: J Am Heart Assoc. 2025 Dec 3;15(1):e044378. doi: 10.1161/JAHA.125.044378 (PMC12909061; doi:10.1161/JAHA.125.044378)
Supplement: Supplementary file 1 — Tables S1–S4 [file JAH3-15-e044378-s001.pdf]

SUPPLEMENTAL MATERIAL

Table S1. Ambulatory physical activity and Heart Rate data per day.

	**Pre-exercise**				**Exercise Day**	**Post-exercise**			
	-4 Days	-3 Days	-2 Days	-1 Day		+1 Day	+2 Days	+3 Days	+4 Days
Sedentary Behaviour minutes (95% CI)	1189 (1140-1238)	1201 (1166-1236)	1176 (1133-1220)	1169 (1117-1221)	1214 (1171-1257)	1198 (1150-1245)	1200 (1160-1240)	1237 (1205-1268)	1173 (1115-1231)
LPA minutes (95% CI)	161 (136-186)	154 (134-175)	174 (150-199)	162 (142-183)	129 (113-146)	157 (139-176)	153 (135-172)	140 (120-160)	147 (127-167)
MVPA minutes (IQR)	54 (41-95)	57* (39-105)	69* (31-138)	63* (39-106)	45 (30-66)	62* (40-87)	62* (35-98)	45 (34-79)	54* (33-114)
Heart Rate beats/min (95% CI)	65* (62-68)	66* (63-68)	65* (62-68)	65* (63-68)	89 (86-93)	67* (64-70)	65* (63-70)	65* (62-68)	66* (63-69)

Sedentary and light physical activity (LPA) time data are presented as mean (95% CI). Moderate-to-vigorous physical activity (MVPA) data are presented as median (IQR).
*Data are significantly different from exercise day ($p < 0.05$). Note: IQR = Interquartile range.

Table S2. Echocardiographic structural and volume characteristics of participants: overall and sex-specific data.

Outcome	Male	n	Female	n	Total	n
Ao R (mm/m^2)	16.5 (15.4-17.6)	12	16.7 (14.6-18.5)	6	16.6 (15.7-17.5)	18
Asc A (mm/m^2)	16.3 (15.0-17.5)	12	16.7 (15.3-18.1)	5	16.4 (15.5-17.3)	17
IVS_d (mm/m^2)	4.73 (4.19-5.27)	12	4.81 (4.32-5.29)	6	4.76 (4.39-5.12)	18
IVC (mm/m^2)	9.96 (8.18-11.7)	11	9.98 (6.12-13.8)	4	9.96 (8.57-11.4)	15
$LVID_d$ (mm/m^2)	24.7 (23.4-25.9)	12	26.2 (21.9-30.4)	6	25.2 (23.8-26.6)	18
$LVID_s$ (mm/m^2)	16.5 (15.4-17.6)	12	17.0 (14.0-20.1)	6	16.7 (15.6-17.7)	18
LV Mass (g/m^2)	119.3 (101.4-137.3)	11	112.9 (84.9-141.0)	6	117.1 (103.7-130.5)	17
LVOT (mm/m^2)	0.107 (0.103-0.112)	12	0.114 (0.097-0.130)	6	0.109 (0.104-0.115)	18
PWT (mm/m^2)	4.75 (4.25-5.25)	11	4.65 (4.07-5.23)	6	4.71 (4.37-5.05)	17
LV EDV (ml/m^2)	67.3 (58.5-76.2)	12	61.1 (43.1-79.2)	6	65.2 (57.8-72.6)	18
LV ESV (ml/m^2)	29.8 (25.6-34.1)	12	24.0 (14.9-33.1)	6	27.9 (24.1-31.7)	18
LAV (ml/m^2)	38.5 (27.7-49.3)	10	38.3 (23.8-52.8)	6	38.4 (30.9-46.0)	16
RAV (ml/m^2)	27.4 (22.8-32.1)	9	31.9 (21.9-41.9)	4	28.8 (25.0-32.6)	13
RV $Length_d$ (mm/m^2)	36.0 (33.3-38.6)	12	37.8 (31.9-43.6)	5	36.5 (34.3-38.7)	17
RV $Basal_d$ (mm/m^2)	21.1 (19.6-22.5)	12	20.3 (16.5-24.1)	6	20.8 (19.5-22.2)	18
RV Mid_d (mm/m^2)	16.6 (15.4-17.9)	12	16.0 (13.7-18.2)	6	16.4 (15.4-17.4)	18
RWT	0.30 (0.25-0.35)	11	0.30 (0.23-0.37)	6	0.30 (0.26-0.34)	17
SV (ml/m^2)	36.7 (29.5-44.0)	8	36.7 (28.2-45.1)	6	36.7 (32.0-41.4)	14

Data are presented as mean [95% CI; number of participants]. Ao R = End-diastolic aortic root diameter; Asc A = Ascending aorta diameter at end diastole; g = Gram; IVC = Inferior vena cava diameter; IVS_d = End-diastolic interventricular septal diameter; LAV = Left atrial volume; LV = Left ventricle; EDV = End diastolic volume; ESV = End systolic volume; $LVID_d$ = Left ventricular internal diameter at end-diastole; $LVID_s$ = Left ventricular internal diameter at end-systole; LV Mass = Left ventricular mass; LVOT = Left ventricular outflow tract diameter at end-diastole; ml = mill; mm = millimetre; m^2 = meter squared; PWT_d = End-diastolic posterior wall thickness; RAV = Right atrial volume; RV = Right ventricle; RV $Basal_d$ = RV basal diameter in diastole; RV $Length_d$ = RV length in diastole; RV Mid_d = RV mid in diastole; RWT = relative wall thickness in diastole; SV = Stroke volume.

Table S3. Resting functional echocardiographic and Sphygmocor vascular characteristics: total sample and sex comparison.

Outcome	Male	n	Female	n	Total	n
2D Echocardiographic characteristics						
AoV V_{max} (m/s)	1.31 (1.22-1.41)	12	1.35 (1.21-1.48)	6	1.32 (1.25-1.39)	18
DT (ms)	212 (196-228)	12	209 (185-234)	6	211 (199-223)	18
EF (%)	56.0 (53.2-58.8)	12	61.2 (54.6-67.7)	6	57.7 (55.0-60.5)	18
E/A ratio	1.46 (1.00-1.91)	12	1.55 (1.22-1.87)	6	1.49 (1.19-1.79)	18
GLS (%)	-13.6 (-2.9 to -24.3)	7	-16.3 (-12.1 to -20.6)	3	-14.4 (-7.6 to -21.2)	10
Lateral E' (cm/s)	12.5 (10.0-15.0)	11	12.6 (10.0-15.2)	5	12.5 (10.8-14.2)	16
LVOT VTI (cm/s)	21.3 (20.4-22.3)	12	20.0 (17.5-22.5)	6	20.9 (20.0-21.8)	18
Peak E (m/s)	0.74 (0.63-0.86)	12	0.85 (0.61-1.09)	6	0.78 (0.68-0.88)	18
Peak A (m/s)	0.58 (0.46-0.69)	12	0.55 (0.48-0.62)	6	0.57 (0.49-0.64)	18
PV V_{max} (m/s)	1.02 (0.82-1.22)	11	0.98 (0.95-1.01)	6	1.01 (0.89-1.13)	17
RV S' (cm/s)	13.1 (11.6-14.6)	12	11.2 (7.6-14.8)	5	12.5 (11.2-13.9)	17
Septal E' (cm/s)	10.2 (8.9-11.4)	12	9.0 (8.1-9.8)	6	9.8 (8.9-10.6)	18
TAPSE (mm)	25.3 (22.7-27.9)	12	24.8 (21.3-28.4)	6	25.2 (23.3-27.1)	18
Tricuspid regurgitation (m/s)*	2.56 (2.38-2.74)	6	2.30	1	2.52 (2.35-2.69)	7
TV V_{max} (m/s)*	0.74 (0.67-0.82)	8	0.80	1	0.75 (0.69-0.81)	9
Sphygmocor characteristics						
BP_s (mmHg)	136.7 (131.8-141.6)	24	125.1 (120.6-129.6)	10	133.2 (129.1-137.2)	34
BP_d (mmHg)	82.3 (79.2-85.3)	24	77.6 (73.1-82.1)	10	80.9 (78.3-83.4)	34
Central BP_s (mmHg)	121.6 (117.1-126.1)	24	114.9 (110.0-119.8)	10	119.6 (116.1-123.0)	34
Central BP_d (mmHg)	83.7 (80.6-86.8)	24	78.5 (74.2-82.8)	10	82.1 (79.5-84.6)	34
HR (bpm)	57.0 (53.6-60.5)	24	57.1 (51.7-62.5)	10	57.1 (54.3-59.8)	34
CF-PWV (m/s)	6.18 (5.76-6.61)	24	5.44 (4.53-6.35)	10	5.96 (5.56-6.35)	34

Data are presented as mean [95% CI]. *Note 3 female and 1 male displayed trivial tricuspid regurgitation, and 2 female and 3 male displayed trivial TV V_{max}, and were not included in the mean value. AoV V_{max} = Aortic valve maximum velocity; BP_d = Blood pressure in diastole; BP_s = Blood pressure in systole; CF-PWV = carotid to femoral pulse wave velocity; DT = mitral valve deceleration time; EF = Ejection fraction; E/A ratio = Ratio of trans mitral flow in early (Peak E) to late (Peak A) diastole; GLS = Global longitudinal strain; HR = Heart rate; Lateral E' = Early diastolic lateral mitral annular relaxation velocity; LVOT VTI = Left ventricular outflow tract velocity time integral; m/s = meters per second; mm = millimetre; Peak E_d = Peak transmitral flow velocity in early diastole; Peak A_d = Peak transmitral flow velocity in late diastole; PV V_{max} = Pulmonary valve maximum velocity; RV S' = Tricuspid annular systolic velocity; Septal E' = Early diastolic septal mitral annular relaxation velocity; TAPSE = Tricuspid annular plane systolic excursion; TV V_{max} = Tricuspid valve maximum velocity.

Table S4. 12-lead ECG findings from 11 participants.

ECG findings	Number of participants (%)
Early repolarisation	6 (55%)
Left ventricular hypertrophy (by voltage)	3 (27%)
Sinus bradycardia	2 (18%)
Left axis deviation	1 (9%)
Poor R wave progression	1 (9%)

Data are presented as the binary count of participants experiencing arrhythmias (yes = 1, no = 0).
